# Impact of COVID-19 Infection on Work Functioning in Japanese Workers: A Prospective Cohort Study

**DOI:** 10.1016/j.shaw.2023.10.004

**Published:** 2023-10-05

**Authors:** Makoto Okawara, Keiki Hirashima, Yu Igarashi, Kosuke Mafune, Keiji Muramatsu, Tomohisa Nagata, Mayumi Tsuji, Akira Ogami, Yoshihisa Fujino, CORoNaWork Project, Akira Ogami, Ayako Hino, Hajime Ando, Hisashi Eguchi, Keiji Muramatsu, Koji Mori, Kosuke Mafune, Makoto Okawara, Mami Kuwamura, Mayumi Tsuji, Ryutaro Matsugaki, Seiichiro Tateishi, Shinya Matsuda, Tomohiro Ishimaru, Tomohisa Nagata, Yoshihisa Fujino, Yu Igarashi

**Affiliations:** 8 The University of Occupational and Environmental Health, Japan; 1 Department of Environmental Epidemiology, Institute of Industrial Ecological Sciences, University of Occupational and Environmental Health, Japan; 2 Disaster Occupational Health Center, Institute of Industrial Ecological Sciences, University of Occupational and Environmental Health, Japan; 3 Department of Mental Health, Institute of Industrial Ecological Sciences, University of Occupational and Environmental Health, Japan; 4 Department of Preventive Medicine and Community Health, School of Medicine, University of Occupational and Environmental Health, Japan; 5 Department of Occupational Health Practice and Management, Institute of Industrial Ecological Sciences, University of Occupational and Environmental Health, Japan; 6 Department of Environmental Health, School of Medicine, University of Occupational and Environmental Health, Japan; 7 Department of Work Systems and Health, Institute of Industrial Ecological Sciences, University of Occupational and Environmental Health, Japan

**Keywords:** COVID-19, occupational health, post-acute COVID-19 syndrome, presenteeism, return to work

## Abstract

**Background:**

The impact of COVID-19 infection on workers' work function persists even after the acute phase of the infection. We studied this phenomenon in Japanese workers.

**Methods:**

We conducted a one-year prospective cohort study online, starting with a baseline survey in December 2020. We tracked workers without baseline work functioning impairment and incorporated data from 14,421 eligible individuals into the analysis. We estimated the incidence rate ratio for new onset of work functioning impairment due to COVID-19 infection during follow-up, using mixed-effects Poisson regression analysis with robust variance.

**Results:**

Participants reporting infection between January and December 2021 showed a significantly higher incidence of new work functioning impairment (adjusted incidence rate ratio: 2.18, 95% confidence interval: 1.75–2.71, *p* < 0.001). The formality of the recuperation environment correlated with a higher risk of work functioning deterioration in infected individuals (*p* for trend <0.001).

**Conclusion:**

COVID-19-infected workers may continue to experience work difficulties due to persistent, post-acute infection symptoms. Companies and society must urgently provide rehabilitation and social support for people with persistent symptoms, recognizing that COVID-19 is not just a transient acute infection.

## 1 Introduction

Beyond its acute infectious effects, COVID-19 exerts extensive long-term health and socioeconomic impacts. Since the 2019 pandemic onset, over 700 million global infections and nearly 7 million fatalities have occurred, primarily within the first two years [1]. Despite the World Health Organization (WHO) ending the Public Health Emergency of International Concern in May 2023, global epidemics and new infections persist [2]. Our COVID-19 response, in a world of continuing socioeconomic activities amidst a steady pandemic, must consider long-term effects alongside approaches to acute-phase disease.

Persistent symptoms following COVID-19 infection pose a challenge. Some individuals may experience enduring symptoms such as fatigue and cognitive dysfunction post-acute SARS-CoV-2 infection [3,4]. Mental health consequences are also noted [5]. These symptoms are collectively known as Long COVID. Although multiple definitions have been reported, a WHO study using the Delphi method defines these conditions as symptoms persisting three months after acute infection and continuing for at least another two months [6]. Some reports have also described cases of fluctuating or shorter-lasting symptoms which do not fit the WHO definition [7]. In the present study, we defined any symptoms continuing after the COVID-19 acute phase as “persistent symptoms,” irrespective of duration.

More than 10% of infected individuals develop persistent symptoms following COVID-19 infection [8,9] and consequent disruption of their social life. Half of patients seeking treatment for these symptoms suffer moderately severe or worse impairment in daily functioning and take at least one day off work per month, while 20% are completely unable to work [8]. Impediments in routine physical activities reduce the quality of life [9]. In one study, half of the hospitalized patients demonstrated cognitive impairment, and 35% experienced dyspnea severe enough to restrict normal activity six months after infection [10]. Many workers still experience symptoms after recovering from COVID-19 and returning to work [11,12].

COVID-19 infection in workers raises concerns about its impact on work function [8,[13], [14], [15]]. Mental Health issues, fatigue, and sleep disorders contribute to work functioning impairment in the workplace. These conditions have also been reported as persistent symptoms [16]. Persistent symptoms such as coughing and fatigue can limit physical work, and decreased cognitive function and mental health symptoms can lead to decreased work ability. The increasing number of workers returning post-COVID-19 infection demands greater attention to their health. However, evidence on the extent of work functioning impairment in Japanese workers post-COVID-19 infection is lacking. We addressed this issue in the present study.

## 2 Methods

We conducted a prospective cohort study using data from the Collaborative Online Research on the Novel-coronavirus and Work (CORoNaWork) Project. The comprehensive design and sampling scheme of the project are detailed elsewhere [17]. Our target population for the online survey consisted of registered monitors of Cross Marketing Inc, which has 4.7 million registrants. Cross Marketing Inc handled the survey operations. All participants received unique IDs, and researchers accessed only anonymized data, allowing the construction of cohort data. The survey company's privacy policy ensured participant information security and prevented researcher access. The study was approved by the ethics committee of the University of Occupational and Environmental Health, Japan.

We carried out the baseline survey from December 22 to 26, 2020. We invited about 600,000 individuals, of whom approximately 55,000 met our eligibility criteria and proceeded to the screening survey. Eligible participants were those aged between 20 and 60, residing in Japan, and employed at the time of the survey. We applied stratified sampling in the screening survey to mitigate bias related to geographical location, job type, and sex. We divided geographical areas into four groups based on the cumulative incidence of COVID-19 at the time of the survey. The area with the highest incidence was further divided into Kanto (Tokyo and surrounding area) and non-Kanto regions, creating a total of five groups. We classified job types as either desk work or non-desk work and sex as men or women. We created 20 stratified units and aimed to balance the number of participants across these units. From this process, we sampled 33,302 individuals. Initially, we excluded 6,266 individuals (19%) who met the predefined exclusion criteria set by the survey company or the researchers. These included very brief response times (less than 6 minutes), unusually short height (less than 140 cm), unusually low weight (less than 30 kg), inconsistent responses to similarly themed questions (e.g., conflicting information about marital status or residence), and incorrect answers to satisficing-detecting questions (e.g., wrongly choosing the third highest number from a group of five). This process yielded 27,036 individuals whose data constituted the baseline for the entire project.

A total of 18,560 individuals (69%) participated in the follow-up survey conducted in December 2021. We excluded individuals with a previous COVID-19 infection at the baseline survey (*n* = 122, 3.2%). We also excluded those experiencing work functioning impairment (Work Functioning Impairment Scale [WFun] score [18] of 21 or higher) at baseline (*n* = 3,790, 20%). Participants who were unemployed at the time of follow-up (*n* = 390, 2.1%) were also excluded. We left out individuals providing inconsistent responses about their COVID-19 infection and recuperation environment at follow-up (*n* = 14, 0.07%). Ultimately, 14,421 individuals were included in the final analysis.

Concerning exposure factors, we used COVID-19 infection experience in the past year, as answered in the follow-up survey. We defined those who answered “yes” to the question: “Have you been diagnosed with COVID-19 infection since January 2021?” as having had a COVID-19 infection during the follow-up period. We excluded participants who reported a COVID-19 infection but who did not answer “yes” to any recuperation questions (i.e., inconsistent responders), as COVID-19 patients in Japan invariably experience one of the following three situations. The three questions about the recuperation environment asked if participants were hospitalized after infection during or after January 2021, if they recuperated at an accommodation facility post-infection, or if they recuperated at home. Based on the responses to these three questions, we classified the patients' treatment experience as follows: 1. not infected, 2. recuperation at home, 3. recuperation at an accommodation facility, or 4. hospitalization. Because it was possible to have multiple recuperation environments depending on the course of symptoms, if “yes” was given in response to more than one question we used the recuperation environment for severe cases. In Japan, hospitalization is the management used for the most severe cases, while home recuperation is used for the mildest cases and those with the least risk of severe disease.

We used various socioeconomic factors as covariates in the baseline survey, including sex, age, and educational background, which ranged from junior high school to graduate school. Marital status options were married, divorced or widowed, and single. We calculated equivalent income from household income divided by the square root of the number of family members. Company size was represented by the number of employees, with the self-employed classified as “1”. We also considered job types, including jobs mainly desk work, jobs mainly involving communication and physical work.

We used the Work Functioning Impairment Scale, a self-reported tool aligned with the consensus-based Standards for the selection of health Measurement Instruments, to assess presenteeism [18]. WFun has been examined for its validity and responsiveness by comparing it with the Stanford presenteeism scale, 8-item Short Form Health Survey, and Work Ability Index [17]. WFun has been also confirmed its convergent validity and responsiveness to changes in the severity of musculoskeletal disorder-related pain and depression [19,20]. The original WFun consists of seven questions, with total scores ranging from 7 to 35. In the baseline survey, we used a six-item version, with scores adjusted based on the Rasch model. We equated moderate to severe work functioning impairment for scores of 21 or more, based on the correlation with evaluations by an occupational health nurse [21]. Participants experiencing this level of impairment at follow-up, but not at baseline, were deemed to have worsening work functioning. Age and WFun scores were treated as continuous variables, using median and interquartile range. We classified WFun scores at baseline into two categories: 7–13 and 14–20, to describe baseline characteristics. Categorical variables were treated as counts and proportions in each category. For participants included in the analysis, we divided equivalent income into quartiles.

We conducted a mixed-effects Poisson regression analysis with robust variance nested within prefectures to estimate the incidence rate ratio (IRR) for deterioration in work functioning due to COVID-19 infection during follow-up. Deterioration of work functioning was the dependent variable, with the occurrence of COVID-19 infection or recuperation environment as independent variables. Model 1 included sex, age, and baseline WFun score as covariates. Along with Model 1 variables, Model 2 included educational background, marital status, equivalent income, company size, and job type as covariates. All analyses were performed using Stata (Stata Statistical Software: Release 17.0; StataCorp LLC, TX), with a p-value of less than 0.05 indicating statistical significance.

## 3 Results

**Fig. 1:**
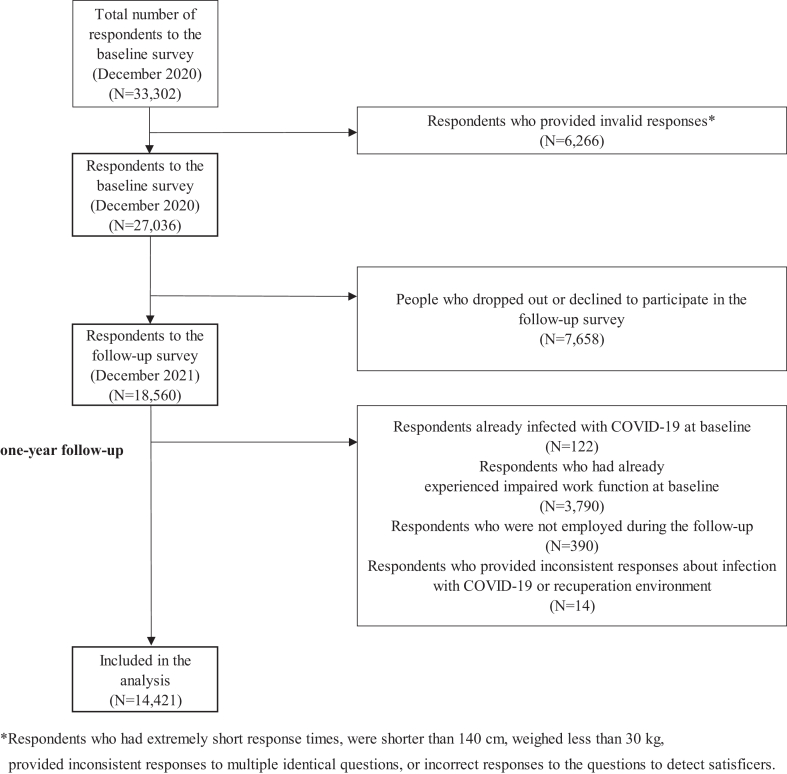
Flow chart of participants in the study.

Fig. 1 shows the flowchart of participants in the study. Of 33,302 respondents, 27,036 respondents were included in the baseline survey, of whom 18,560 (69%) responded to the follow-up survey. Ultimately, 14,421 were incorporated into the analysis.

Table 1 displays the baseline characteristics of participants. Infected individuals included a higher proportion of men and fewer desk-work workers than non-infected ones. Hospitalization was the most common recuperation environment for infected individuals, at 42.9%.

**Table 1:** Baseline characteristics according to the experience of infection of COVID-19 during the follow-up period

	Included in the analysis		
	Total	Experience of infection of COVID-19 during the follow-up period	
		No	Yes
	N = 14,421	N = 14,253	N = 168
	n (%)	n (%)	n (%)
Sex, men	8,324 (57.7)	8,215 (57.6)	109 (64.9)
Age, years, median (interquartile range)	50 (43-57)	50 (43-57)	49 (40-56)
Education			
Junior high school	191 (1.3)	190 (1.3)	1 (0.6)
High school	3,750 (26.0)	3,705 (26.0)	45 (26.8)
Vocational school, junior college, or technical college	3,292 (22.8)	3,249 (22.8)	43 (25.6)
University or graduate school	7,188 (49.8)	7,109 (49.9)	79 (47.0)
Marital status			
Married	8,328 (57.7)	8,232 (57.8)	96 (57.1)
Divorced or widowed	1,472 (10.2)	1,456 (10.2)	16 (9.5)
Never married	4,621 (32.0)	4,565 (32.0)	56 (33.3)
Equivalent income (Japanese Yen)			
-2,500,000	3,353 (23.3)	3,313 (23.2)	40 (23.8)
2,500,000–3,800,000	3,826 (26.5)	3,774 (26.5)	52 (31.0)
3,800,000–5,250,000	3,132 (21.7)	3,099 (21.7)	33 (19.6)
5,250,000-	4,110 (28.5)	4,067 (28.5)	43 (25.6)
Job type			
Mainly desk work	7,398 (51.3)	7,320 (51.4)	78 (46.4)
Jobs mainly involving interpersonal communication	3,556 (24.7)	3,511 (24.6)	45 (26.8)
Mainly physical work	3,467 (24.0)	3,422 (24.0)	45 (26.8)
Number of employees			
1	1,479 (10.3)	1,469 (10.3)	10 (6.0)
2-49	4,423 (30.7)	4,370 (30.7)	53 (31.5)
50-999	4,939 (34.2)	4,875 (34.2)	64 (38.1)
1,000–9,999	2,436 (16.9)	2,408 (16.9)	28 (16.7)
≥10,000	1,144 (7.9)	1,131 (7.9)	13 (7.7)
WFun total score at baseline∗, median (interquartile range)	7 (7-13)	7 (7-13)	8.5 (7-14)
7-13	10,985 (76.2)	10,870 (76.3)	115 (68.5)
14-20	3,436 (23.8)	3,383 (23.7)	53 (31.5)
Procedure of recuperation when infected with COVID-19			
Not infected	14,253 (98.8)	14,253 (100)	0 (0)
Recuperation at home	56 (0.4)	0 (0)	56 (33.3)
Recuperation at accommodation facility	40 (0.3)	0 (0)	40 (23.8)
Hospitalization	72 (0.5)	0 (0)	72 (42.9)

WFun: The Work Functioning Impairment Scale.

∗ WFun scores from 7 to 35 points, with 21 or more points being excluded.

Table 2 connects the onset of work functioning impairment at follow-up with the presence of COVID-19 infection or the recuperation environment used during infection. Participants who reported infection between January and December 2021 presented higher WFun scores and incidence of work functioning impairment. The mixed-effects Poisson regression analysis with robust variance revealed significantly higher IRR for the onset of work functioning impairment among infected individuals in Model 1 and Model 2 (adjusted IRR: 2.18, 95% confidence interval [CI]: 1.75–2.71, *p* < 0.001). WFun scores at follow-up and incidence of work functioning impairment increased in the order of uninfected participants, those who were infected and recuperated at home, those who recuperated at an accommodation facility, and those who were hospitalized. Using uninfected participants as a reference, IRR for the onset of work functioning impairment rose with more severe recuperation environments in both Models 1 and 2 (*p* for trend <0.001).

**Table 2:** Association between experience of infection of COVID-19 and worsening of work function

	WFun score at the follow-up	Worsening of work function∗	Model 1				Model 2			
Variable	Median (interquartile range)	%	Incidence rate ratio	95% confidence interval		*p*	Incidence rate ratio	95% confidence interval		*p*
Experience of infection of COVID-19 during the follow-up period										
No	11 (7-16)	14	reference				reference			
Yes	16 (9-23)	36	2.20	1.77	2.74	<0.001	2.18	1.75	2.71	<0.001
Procedure of recuperation										
Not infected	11 (7-16)	14	reference			<0.001†	reference			<0.001†
Recuperation at home	14 (8-20)	23	1.37	0.75	2.51	0.304	1.34	0.75	2.41	0.320
Recuperation at accommodation facility	17 (11-21.5)	28	1.76	1.12	2.77	0.014	1.71	1.09	2.67	0.019
Hospitalization	21 (9.5-25.5)	51	3.09	2.45	3.90	<0.001	3.00	2.40	3.75	<0.001

WFun: The Work Functioning Impairment Scale.

Model 1: adjusted for sex, age, and WFun score at baseline.

Model 2: adjusted for sex, age, WFun score at baseline, education, marital status, equivalent income, company size, job type.

∗ worsening to a score of 21 or more on The Work Functioning Impairment Scale at follow-up.

† *p* for trend.

We conducted a sensitivity analysis using the same statistical models as the main analysis, with multiple cut-offs of 24 points and 28 points. We also conducted mixed-effects linear regression using differences in WFun scores at baseline and follow-up as outcomes. All analyses showed similar results.

## 4 Discussion

This study investigated the association of COVID-19 infection during follow-up with baseline work functioning deterioration, based on one-year prospective cohort data. The results revealed that workers with COVID-19 infection during follow-up experienced significant work functioning deterioration compared to non-infected workers.

Work functioning impairment arises when job demands mismatch with a worker's symptoms or condition, for example, when concentration declines due to mental health issues. The productivity decrease from work functioning impairment is termed presenteeism [22]. Various physical, mental, and cognitive symptoms may appear in people infected with COVID-19, potentially leading to prolonged work functioning impairment. This situation is highlighted by the gap between treatment and recuperation duration and symptom duration. Confirmed COVID-19 infection typically results in a recommended 5–20-day post-infection isolation period, depending on symptom severity [23,24]. However, some individuals exhibit symptoms persisting beyond the required public health isolation period.

Several reasons may be proposed for why COVID-19-infected individuals experience work functioning impairment. First, detrimental mental health effects occur following COVID-19 infection, at least in the short-term. One month post-hospital discharge, patients demonstrate a high prevalence of depression and PTSD symptoms [25]. Mental health problems are a well-known cause of work functioning impairment [16,26]. However, we suggest that work functioning impairment causes extend beyond mental health issues to include physical symptom-based factors. This argument is supported by the persistence of the significantly increased risk after preliminary adjustment for mental distress at follow-up using the K6 score.

Second, persistent cognitive dysfunction after COVID-19 infection is an important symptom. “Brain fog,” a typical symptom, signifies declines in concentration, attention, and memory [27]. Evidence exists for central nervous system sequelae following SARS-CoV-2 infection [28,29]. Reports show that one in five people experience cognitive dysfunction more than 12 weeks after COVID-19 diagnosis [30]; indeed, in one study, cognitive impairment was observed in 38% of patients at four months post-discharge [31].

Third, persistent fatigue is commonly associated with workers' performance in various settings. Reports show that 32% of people experience fatigue more than 12 weeks post-COVID-19 diagnosis [30] and that fatigue is the main cause of functional impairment in those treated for persistent symptoms [29]. Working on physical or intellectual tasks while fatigued can lead to functional impairment due to inadequate performance.

Fourth, shortness of breath directly affects physical work function. Surveys 3–4 months post-COVID-19 discharge show that 16% of people experienced difficulty breathing, and 21% of people with any new-onset symptom except anosmia showed objective respiratory failure [31]. This symptom poses particular problems in physical work requiring a high degree of physical exertion.

This study revealed differential risks for work functioning impairment among COVID-19 patients in Japan, depending on their recuperation environment. The recuperation environment in Japan reflects COVID-19 severity to some extent, and the severity of acute infection has been identified as a risk for persistent symptoms [32,33]. Three recuperation practices are commonly used for COVID-19 patients in Japan. During the pandemic, the statutory standard approach involved hospitalization for isolation and treatment. When patient numbers surged beyond hospital capacity, doctors treated and monitored those showing less severe symptoms at accommodation facilities. Low-risk or nearly asymptomatic patients recuperated at home under public health center monitoring.

Companies must check for functional impairment in workers who have contracted COVID-19 and provide them with additional support if necessary. Persistent symptoms of cognitive dysfunction may significantly impact job performance and safety and, therefore, require attention. To support workers suffering from fatigue, measures such as improving workplace rest spaces, implementing flexible working hours, and expanding work-from-home options can be considered. For workers with shortness of breath, it may be necessary to consider job reassignment, along with allowing more flexibility for breaks and facilitating sharing of information to promote understanding among colleagues [31]. Consideration is being given to support measures for COVID-19 patients returning to work, taking into account symptom and task combinations [32]. Instead of viewing COVID-19 as a transient acute infectious disease, we urgently need to provide rehabilitation and social support for persistent symptoms.

This study has several limitations. First, self-reported COVID-19 infection experience may cause misclassification. However, due to the pandemic's significance, respondents are unlikely to forget or misreport their COVID-19 infection. Moreover, misclassification usually attenuates any reported association, which could originally have been stronger. Second, the concurrent collection of infection experience and work functioning impairment data during follow-up introduce potential recall bias. Individuals with work functioning impairment may be more likely to remember their COVID-19 infection. However, those without work functioning impairment are unlikely to forget or intentionally omit to report past-year COVID-19 infection. Third, individuals with persistent post-COVID-19 symptoms and social difficulties may have been more likely to drop out of the follow-up. If so, the actual association might be stronger than the results suggest. Fourth, as the precise timing of infection was not specified, it is unclear if work functioning impairment was due to acute recovery phase symptoms or persistent “Long COVID”. The effects of long-term COVID-19 on anxiety, depression, and sleep appear insignificant [34]. Reports suggest that PTSD symptoms diminish over time [25]. Finally, as we did not identify persistent symptoms in individual workers, we were unable to examine specific symptom impacts on work functioning impairment, or explore symptom-job type combinations.

In conclusion, among unimpaired workers, COVID-19 infection significantly increased the risk of new work functioning impairment over a one-year follow-up. As COVID-19 persists, it becomes critical to establish causes and treatments for persisting symptoms and devise appropriate responses for affected workers.

## Conflicts of interest

Dr. Fujino holds the copyright to WFun with royalties paid from Sompo Health Support Inc., outside of this work. The other authors declare no conflicts of interest associated with this manuscript.This study was supported and partly funded by a research grant from the University of Occupational and Environmental Health, Japan (no grant number); Japanese Ministry of Health, Labor and Welfare (H30-josei-ippan-002, H30-roudou-ippan-007, 19JA1004, 20JA1006, 210,301-1, and 20HB1004); Anshin Zaidan (no grant number), the Collabo-Health Study Group (no grant number) and Hitachi Systems, Ltd. (no grant number), and scholarship donations from Chugai Pharmaceutical Co., Ltd. (no grant number). No funder was involved in the study design, collection, analysis, interpretation of data, the writing of this article or the decision to submit it for publication.

## Declaration of generative AI and AI-assisted technologies in the writing process

No single sentence or ideas were derived from generative AI. We used ChatGPT (OpenAI, San Francisco, California, United States), only to enhance this paper's English quality, followed by checks from the authors and a professional English editing company.
